# Supplementary figures and images for: Nearly half of adults with symptoms of sexually transmitted infections (STIs) did not seek clinical care: A population-based study of treatment-seeking behavior among adults in Rakai, Uganda
Source: PLOS Glob Public Health. 2023 May 1;3(5):e0001626. doi: 10.1371/journal.pgph.0001626 (PMC10150988; doi:10.1371/journal.pgph.0001626)

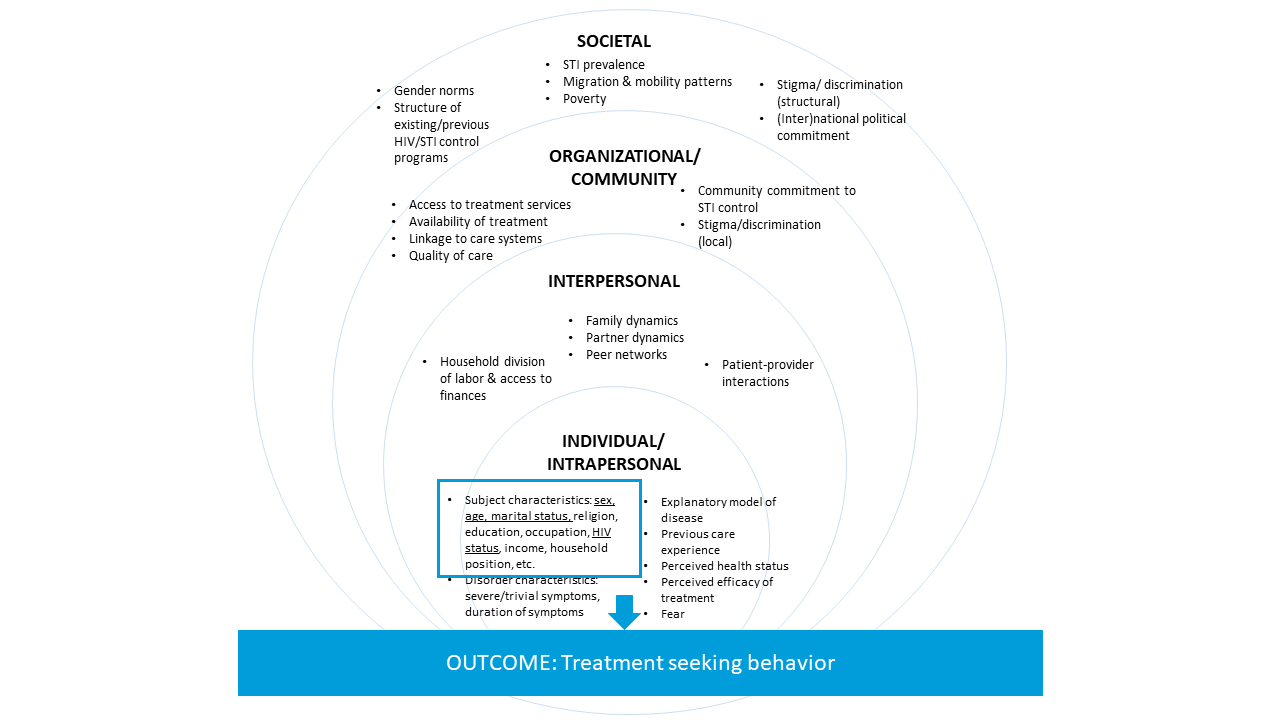

Supplement: S1 Fig — Informed by Kroeger’s determinants model and McLeroy et al.’s socioecological framework. This conceptual framework was developed to guide statistical analyses. Variables selected for inclusion in the multivariate model are underlined. (TIF) [file pgph.0001626.s001.TIF]
