# Supplementary material for: Nearly half of adults with symptoms of sexually transmitted infections (STIs) did not seek clinical care: A population-based study of treatment-seeking behavior among adults in Rakai, Uganda
Source: PLOS Glob Public Health. 2023 May 1;3(5):e0001626. doi: 10.1371/journal.pgph.0001626 (PMC10150988; doi:10.1371/journal.pgph.0001626)
Supplement: S1 Table — Data are presented as n (%). (DOCX) [file pgph.0001626.s002.docx]

**S1 Table.** **Prevalence of private and government clinic treatment seeking among STIPS participants who reported seeking clinic treatment for STI symptoms in the past 6 months (N=450), by sex and community type. Data are presented as n (%).**

A – By community type

|  |  | **Sex** | **Crude PRR**  **(95% CI)** |
| --- | --- | --- | --- |
| **Community** | **Treatment Location** | **Female N=315** |  |
| Inland (N=197) | Government | 78/93 (84%) | REF |
|  | Private | 67/104 (64%) | 0.65*** (0.51-0.83) |
| Fishing (N=253) | Government | 40/49 (82%) | REF |
|  | Private | 130/204 (64%) | 0.86*** (0.77-0.96) |
| *** p≤0.01, ** p≤0.05, * p≤0.1 | | | |

B – By sex

|  |  | **Community Type** | **Crude PRR**  **(95% CI)** |
| --- | --- | --- | --- |
| **Sex** | **Treatment Location** | **Fishing N=253** |  |
| Male (N=135) | Government | 9/24 (38%) | REF |
|  | Private | 74/111 (67%) | 1.25** (1.04-1.51) |
| Female (N=315) | Government | 40/118 (34%) | REF |
|  | Private | 130/197 (66%) | 1.65*** (1.36-2.01) |
| *** p≤0.01, ** p≤0.05, * p≤0.1 | | | |
