# Supplementary material for: Nearly half of adults with symptoms of sexually transmitted infections (STIs) did not seek clinical care: A population-based study of treatment-seeking behavior among adults in Rakai, Uganda
Source: PLOS Glob Public Health. 2023 May 1;3(5):e0001626. doi: 10.1371/journal.pgph.0001626 (PMC10150988; doi:10.1371/journal.pgph.0001626)
Supplement: S2 Table — Data are presented as n (%). (DOCX) [file pgph.0001626.s003.docx]

**S2. Table. Unadjusted prevalence of clinic treatment seeking among STIPS participants who reported STI symptoms in the past 6 months (N=956), for the full sample. Data are presented as n (%).**

| **FULL SAMPLE (N=956)** | | |
| --- | --- | --- |
|  | **Sought Clinic Treatment** | **Crude PRR**  **(95% CI)** |
|  | **Yes**  **N=457** |  |
| Sex |  |  |
| Male | 136/288 (47%) | REF |
| Female | 321/668 (48%) | 1.02 (0.88-1.18) |
| Age |  |  |
| 15-19 years | 30/69 (43%) | REF |
| 20-29 years | 186/368 (51%) | 1.16 (0.87-1.55) |
| 30-39 years | 171/355 (48%) | 1.11 (0.83-1.48) |
| 40-49 years | 70/164 (43%) | 0.98 (0.71-1.36) |
| Marital status |  |  |
| Never Married | 30/73 (41%) | REF |
| Married, Monogamous | 262/558 (47%) | 1.14 (0.86-1.52) |
| Married, Polygamous | 64/127 (50%) | 1.23 (0.89-1.70) |
| Previously Married | 101/198 (51%) | 1.24 (0.91-1.69) |
| Community type |  |  |
| Inland | 202/418 (48%) | REF |
| Fishing | 255/538 (47%) | 0.98 (0.86-1.12) |
| Religion |  |  |
| Christian | 388/802 (48%) | REF |
| Muslim | 57/122 (47%) | 0.97 (0.79-1.18) |
| Other/none | 3/6 (50%) | 1.03 (0.46-2.31) |
| Education |  |  |
| None | 25/62 (40%) | REF |
| Some primary education or above | 432/894 (48%) | 1.20 (0.88-1.63) |
| Occupation |  |  |
| Agricultural or housework | 165/342 (48%) | REF |
| Bar or restaurant work | 32/70 (46%) | 0.95 (0.72-1.25) |
| Boda boda driving or trucking | 9/19 (47%) | 0.98 (0.60-1.60) |
| Fishing | 51/127 (40%) | 0.83 (0.66-1.06) |
| Student | 5/12 (42%) | 0.86 (0.44-1.70) |
| Trader or shopkeeper | 106/219 (48%) | 1.00 (0.84-1.20) |
| Other | 89/167 (53%) | 1.10 (0.92-1.32) |
| HIV status |  |  |
| Negative | 298/645 (46%) | REF |
| Positive | 157/309 (51%) | 1.10 (0.96-1.26) |
| Sex in the past year |  |  |
| No | 18/54 (33%) | REF |
| Yes | 439/902 (49%) | 1.46** (1.00-2.14) |
| Sexual partners in the past year |  |  |
| None | 18/54 (33%) | 0.68** (0.47-1.01) |
| 1 | 306/628 (49%) | REF |
| 2-4 | 115/242 (48%) | 0.98 (0.84-1.14) |
| 5-10 | 13/24 (54%) | 1.11 (0.76-1.62) |
| >10 | 5/8 (62%) | 1.28 (0.75-2.21) |
| Sex with partner from outside the community |  |  |
| No | 343/708 (48%) | REF |
| Yes | 114/248 (46%) | 0.95 (0.81-1.11) |
| Lifetime sexual partners |  |  |
| None | 1/10 (10%) | 0.21* (0.03-1.37) |
| 1 | 32/77 (42%) | 0.88 (0.66-1.17) |
| 2-4 | 216/458 (47%) | REF |
| 5-10 | 192/372 (52%) | 1.09 (0.95-1.26) |
| >10 | 16/39 (41%) | 0.87 (0.59-1.28) |
| Condom use in past 12 months |  |  |
| Marital partners only | 259/544 (48%) | REF |
| Non-marital partners, inconsistent or no use | 162/325 (50%) | 1.06 (0.92-1.21) |
| Non-marital partners, consistent use | 30/61 (49%) | 1.03 (0.79-1.35) |
| NA, no sexual partners in past 12 months | 6/32 (19%) | 0.39** (0.19-0.81) |
| Number of STI symptoms in past 6 months |  |  |
| 1 | 112/312 (36%) | REF |
| 2-4 | 230/457 (50%) | 1.40*** (1.18-1.67) |
| >=5 | 115/187 (61%) | 1.71*** (1.42-2.06) |
| Symptoms in the past 7 days |  |  |
| No | 183/353 (52%) | REF |
| Yes | 274/603 (45%) | 0.88** (0.77-1.00) |
| Number of STI symptoms in past 7 days |  |  |
| 0 | 183/353 (52%) | REF |
| 1 | 100/260 (38%) | 0.74*** (0.62-0.89) |
| 2-4 | 121/249 (49%) | 0.94 (0.80-1.10) |
| >=5 | 53/94 (56%) | 1.09 (0.89-1.33) |
| Genital ulcer |  |  |
| No | 295/675 (44%) | REF |
| Yes | 162/281 (58%) | 1.32*** (1.16-1.51) |
| Genital discharge |  |  |
| No | 210/495 (42%) | REF |
| Yes | 247/461 (54%) | 1.26*** (1.11-1.44) |
| Thick and/or colored vaginal discharge |  |  |
| No | 296/638 (46%) | REF |
| Yes | 161/318 (51%) | 1.09 (0.95-1.25) |
| Itching of the vagina |  |  |
| No | 236/539 (44%) | REF |
| Yes | 221/417 (53%) | 1.21*** (1.06-1.38) |
| Unpleasant vaginal odor |  |  |
| No | 363/774 (47%) | REF |
| Yes | 94/182 (52%) | 1.10 (0.94-1.29) |
| Frequent urination |  |  |
| No | 343/733 (47%) | REF |
| Yes | 114/223 (51%) | 1.09 (0.94-1.27) |
| Painful urination |  |  |
| No | 287/687 (42%) | REF |
| Yes | 170/269 (63%) | 1.51*** (1.33-1.72) |
| Pain during intercourse |  |  |
| No | 370/806 (46%) | REF |
| Yes | 87/150 (58%) | 1.26*** (1.08-1.48) |
| Bleeding during intercourse |  |  |
| No | 453/941 (48%) | REF |
| Yes | 4/15 (27%) | 0.55 (0.24-1.29) |
| Lower abdominal pain |  |  |
| No | 259/577 (45%) | REF |
| Yes | 198/379 (52%) | 1.16** (1.02-1.33) |
| Genital warts |  |  |
| No | 443/906 (49%) | REF |
| Yes | 14/50 (28%) | 0.57** (0.37-0.90) |
| *** p≤0.01, ** p≤0.05, * p≤0.1, PRR: prevalence rate ratio | | |
