# Supplementary material for: Nearly half of adults with symptoms of sexually transmitted infections (STIs) did not seek clinical care: A population-based study of treatment-seeking behavior among adults in Rakai, Uganda
Source: PLOS Glob Public Health. 2023 May 1;3(5):e0001626. doi: 10.1371/journal.pgph.0001626 (PMC10150988; doi:10.1371/journal.pgph.0001626)
Supplement: S3 Table — Data are presented as n (%). (DOCX) [file pgph.0001626.s004.docx]

**S3. Table. Unadjusted prevalence of clinic treatment seeking among STIPS participants who reported STI symptoms in the past 6 months (N=956), sociodemographic characteristics and behaviors by sex. Data are presented as n (%).**

|  | **MALES (N=288)** | | **FEMALES (N=668)** | |
| --- | --- | --- | --- | --- |
|  | **Sought Clinic Treatment** |  | **Sought Clinic Treatment** |  |
|  | **Yes**  **N=136** | **Crude PRR**  **(95% CI)** | **Yes**  **N=321** | **Crude PRR**  **(95% CI)** |
| Age |  |  |  |  |
| 15-19 years | 6/14 (43%) | REF | 24/55 (44%) | REF |
| 20-29 years | 52/97 (54%) | 1.25 (0.66-2.36) | 134/271 (49%) | 1.13 (0.82-1.57) |
| 30-39 years | 52/114 (46%) | 1.06 (0.56-2.02) | 119/241 (49%) | 1.13 (0.82-1.57) |
| 40-49 years | 26/63 (41%) | 0.96 (0.49-1.89) | 44/101 (44%) | 1.00 (0.69-1.45) |
| Marital status |  |  |  |  |
| Never Married | 17/32 (53%) | REF | 13/41 (32%) | REF |
| Married, Monogamous | 71/173 (41%) | 0.77 (0.53-1.12) | 191/385 (50%) | 1.56* (0.99-2.48) |
| Married, Polygamous | 10/23 (43%) | 0.82 (0.46-1.45) | 54/104 (52%) | 1.64** (1.01-2.66) |
| Previously Married | 38/60 (63%) | 1.19 (0.82-1.74) | 63/138 (46%) | 1.44 (0.89-2.34) |
| Community type |  |  |  |  |
| Inland | 53/96 (55%) | REF | 149/322 (46%) | REF |
| Fishing | 83/192 (43%) | 0.78** (0.61-1.00) | 172/346 (50%) | 1.07 (0.92-1.26) |
| Religion |  |  |  |  |
| Christian | 119/242 (49%) | REF | 269/560 (48%) | REF |
| Muslim | 16/45 (36%) | 0.72 (0.48-1.09) | 41/77 (53%) | 1.11 (0.88-1.39) |
| Other/none | 1/1 (100%) | 2.03*** (1.79-2.31) | 2/5 (40%) | 0.83 (0.28-2.45) |
| Education |  |  |  |  |
| None | 10/21 (48%) | REF | 15/41 (37%) | REF |
| Some primary education or  above | 126/267 (47%) | 0.99 (0.62-1.58) | 306/627 (49%) | 1.33 (0.88-2.01) |
| Occupation |  |  |  |  |
| Agricultural or housework | 29/50 (58%) | REF | 136/292 (47%) | REF |
| Bar or restaurant work | 0/1 (0%) | - | 32/69 (46%) | 1.00 (0.75-1.32) |
| Boda boda driving or  trucking | 9/19 (47%) | 0.82 (0.48-1.39) | 0/0 (0%) | - |
| Fishing | 51/127 (40%) | 0.69** (0.50-0.95) | 0/0 (0%) | - |
| Student | 2/4 (50%) | 0.86 (0.31-2.37) | 3/8 (38%) | 0.81 (0.33-1.99) |
| Trader or shopkeeper | 17/36 (47%) | 0.81 (0.54-1.24) | 89/183 (49%) | 1.04 (0.86-1.27) |
| Other | 28/51 (55%) | 0.95 (0.67-1.33) | 61/116 (53%) | 1.13 (0.91-1.40) |
| HIV status |  |  |  |  |
| Negative | 91/199 (46%) | REF | 207/446 (46%) | REF |
| Positive | 45/89 (51%) | 1.11 (0.86-1.43) | 112/220 (51%) | 1.10 (0.93-1.29) |
| Sex in the past year |  |  |  |  |
| No | 5/9 (56%) | REF | 13/45 (29%) | REF |
| Yes | 131/279 (47%) | 0.85 (0.46-1.54) | 308/623 (49%) | 1.71** (1.07-2.73) |
| Sexual partners in the past year |  |  |  |  |
| None | 5/9 (56%) | 1.21 (0.65-2.25) | 13/45 (29%) | 0.59** (0.37-0.94) |
| 1 | 47/102 (46%) | REF | 259/526 (49%) | REF |
| 2-4 | 68/147 (46%) | 1.00 (0.76-1.32) | 47/95 (49%) | 1.00 (0.81-1.25) |
| 5-10 | 12/23 (52%) | 1.13 (0.73-1.77) | 1/1 (100%) | 2.03*** (1.86-2.22) |
| >10 | 4/7 (57%) | 1.24 (0.63-2.44) | 1/1 (100%) | 2.03*** (1.86-2.22) |
| Sex with partner from outside the community |  |  |  |  |
| No | 76/162 (47%) | REF | 267/546 (49%) | REF |
| Yes | 60/126 (48%) | 1.02 (0.79-1.30) | 54/122 (44%) | 0.91 (0.73-1.12) |
| Lifetime sexual partners |  |  |  |  |
| None | 0/2 (0%) | - | 1/8 (12%) | 0.26 (0.04-1.65) |
| 1 | 1/1 (100%) | 2.26*** (1.67-3.07) | 31/76 (41%) | 0.86 (0.64-1.15) |
| 2-4 | 23/52 (44%) | REF | 193/406 (48%) | REF |
| 5-10 | 99/203 (49%) | 1.10 (0.79-1.54) | 93/169 (55%) | 1.16* (0.98-1.37) |
| >10 | 13/30 (43%) | 0.98 (0.59-1.63) | 3/9 (33%) | 0.70 (0.28-1.78) |
| Condom use in past 12 months |  |  |  |  |
| Marital partners only | 43/102 (42%) | REF | 216/440 (49%) | REF |
| Non-marital partners,  inconsistent or no use | 80/150 (53%) | 1.27* (0.96-1.66) | 82/171 (48%) | 0.98 (0.81-1.17) |
| Non-marital partners,  consistent use | 12/31 (39%) | 0.92 (0.56-1.51) | 18/30 (60%) | 1.22 (0.90-1.66) |
| NA, no sexual partners in  past 12 months | 1/5 (20%) | 0.47 (0.08-2.79) | 5/27 (19%) | 0.38** (0.17-0.84) |
| *** p≤0.01, ** p≤0.05, * p≤0.1; NA: Not applicable | | | | |
