# Supplementary material for: Nearly half of adults with symptoms of sexually transmitted infections (STIs) did not seek clinical care: A population-based study of treatment-seeking behavior among adults in Rakai, Uganda
Source: PLOS Glob Public Health. 2023 May 1;3(5):e0001626. doi: 10.1371/journal.pgph.0001626 (PMC10150988; doi:10.1371/journal.pgph.0001626)
Supplement: S4 Table — (DOCX) [file pgph.0001626.s005.docx]

**S4 Table. Predictors of clinic treatment seeking among STIPS participants who reported STI symptoms in the past 6 months (N=956), by sex**

|  | **MALES** | **FEMALES** |
| --- | --- | --- |
|  | **Adjusted PRR**  **(95% CI)** | **Adjusted PRR**  **(95% CI)** |
| Age |  |  |
| 15-19 years | REF | REF |
| 20-29 years | 1.10 (0.61-2.00) | 0.97 (0.70-1.33) |
| 30-39 years | 1.01 (0.54-1.90) | 0.96 (0.69-1.33) |
| 40-49 years | 0.85 (0.44-1.64) | 0.89 (0.61-1.31) |
| Community type |  |  |
| Inland | REF | REF |
| Fishing | 0.70*** (0.55-0.89) | 0.97 (0.82-1.15) |
| Marital status |  |  |
| Never Married | REF | REF |
| Married, Monogamous | 0.92 (0.61-1.37) | 1.57* (0.96-2.58) |
| Married, Polygamous | 0.91 (0.52-1.59) | 1.67* (0.99-2.81) |
| Previously Married | 1.44* (0.97-2.14) | 1.46 (0.86-2.47) |
| HIV status |  |  |
| Negative | REF | REF |
| Positive | 1.27* (0.98-1.67) | 1.08 (0.90-1.30) |
| Number of STI symptoms in past 6 months |  |  |
| 1 | REF | REF |
| >1 | 1.73*** (1.36-2.21) | 1.41*** (1.12-1.78) |
| *** p≤0.01, ** p≤0.05, * p≤0.1 | | |
