# Supplementary material for: Nearly half of adults with symptoms of sexually transmitted infections (STIs) did not seek clinical care: A population-based study of treatment-seeking behavior among adults in Rakai, Uganda
Source: PLOS Glob Public Health. 2023 May 1;3(5):e0001626. doi: 10.1371/journal.pgph.0001626 (PMC10150988; doi:10.1371/journal.pgph.0001626)
Supplement: Attachment — Submitted filename: 2023.03.27_Ogale_STI Txt Seeking_Response to reviewers.docx [file pgph.0001626.s006.docx]

March 27, 2023

Dear Editors,

Thank you for the opportunity to revise and re-submit our manuscript “Nearly half of adults with symptoms of sexually transmitted infections (STIs) did not seek clinical care: A population-based study of treatment-seeking behavior among adults in Rakai, Uganda” in consideration of publication in *PLOS Global Public Health*.

We appreciate the reviewers’ thoughtful comments and suggestions. We have copied the comments and addressed each of them below, describing any corresponding edits to the manuscript. The location in the revised manuscript has been identified using the line numbers in the tracked changes version. We think these edits have strengthened the manuscript and look forward to working with you to prepare manuscript appropriate to publish in *PLOS Global Public Health*.

Thank you for your consideration,

Yasmin P. Ogale

Yasmin.ogale@gmail.com

| **Reviewer comments** | **Response** | **Related edits**  **(Line numbers based on the track changes version)** |
| --- | --- | --- |
| ***Journal Requirements*** | | |
| Please review your reference list to ensure that it is complete and correct. If you have cited papers that have been retracted, please include the rationale for doing so in the manuscript text, or remove these references and replace them with relevant current references. Any changes to the reference list should be mentioned in the rebuttal letter that accompanies your revised manuscript. If you need to cite a retracted article, indicate the article’s retracted status in the References list and also include a citation and full reference for the retraction notice. | We have reviewed the reference list. There are no cited papers that have been retracted. | N/A |
| Please amend your detailed Financial Disclosure statement. This is published with the article. It must therefore be completed in full sentences and contain the exact wording you wish to be published.  a. State the initials, alongside each funding source, of each author to receive each grant. b. State what role the funders took in the study. If the funders had no role in your study, please state: “The funders had no role in study design, data collection and analysis, decision to publish, or preparation of the manuscript.” c. If any authors received a salary from any of your funders, please state which authors and which funders.  If you did not receive any funding for this study, please simply state: “The authors received no specific funding for this work.” | We have amended our Financial Disclosure Statement. | This work was supported by grants from the Johns Hopkins Center for AIDS Research (**MKG**; grant number P30AI094189), grants from the National Institute of Allergy and Infectious Diseases (**MKG**; grant numbers R01AI143333, R01MH115799, and K01AI125086), the National Institute of Mental Health (**MKG**; grant number R01MH107275), the Eunice Kennedy Shriver National Institute of Child Health and Human Development (**MKG**; grant number RO1HD091003), and the Division of Intramural Research of the National Institute for Allergy and Infectious Diseases (**MKG**). **The funders had no role in study design, data collection and analysis, decision to publish, or preparation of the manuscript.** |
| Please provide separate figure files in .tif or .eps format only and remove any figures embedded in your manuscript file. Please also ensure that all files are under our size limit of 10MB.  For more information about figure files please see our guidelines:  <https://journals.plos.org/globalpublichealth/s/figures>  <https://journals.plos.org/globalpublichealth/s/figures#loc-file-requirement> | We have provided separate figure files in .tif format. We have also added legends for figures in line with the text. | Uploaded as separate files. Legends on lines 276-279, 312-316. |
| We have noticed that you have uploaded Supporting Information files, but you have not included a list of legends. Please add a full list of legends for your Supporting Information files after the references list. | We have added a list of legends for supporting information files after the references list. | Lines 860-881. |
| ***Reviewer #1*** |  |  |
| The manuscript by Ogale et al. presents important information on treatment seeking behaviour for STIs in Uganda. As discussed by the authors, this is a relatively under-researched area as most studies enrol participants when they attend for care. The introduction was informative and concise, and the methodology appears to be sound and was generally very clear, although more information should be provided on the conceptual framework underlying the multivariate analysis. Although the results section was adequately presented, I did find the large number of analyses quite overwhelming. Although I understand the rationale behind presenting results for male and female participants separately, the reader has to process double the number of results, and this should be taken into consideration when deciding on which results to present. At present, the results section is very long. Despite this, the discussion was thorough and did an excellent job of summarising the results and placing it in the context of the literature, particularly regarding HIV and fishing communities, as well as some of the limitations of the study.  Overall, I think the results section could be made more concise. Otherwise, I have suggested mostly minor edits for clarity. | Thank you for the comments and commendation! We agree that the results section can be made more concise. In addition to the revisions described below, which include adding more information on the underlying conceptual framework, we have also moved some of the analyses to the supplement so as not to overwhelm the reader. We hope you agree that the results section is more focused after these edits. | We moved Table 2 data for the full sample from Table 2 to S2 Table, and moved demographic and behavioral data for men and women to S3 Table. We also made additional changes throughout the Results section, as documented in track changes and detailed below. |
| 1. Lines 95-102: “Previous studies in the region have also found gender differences in treatment-seeking, with barriers to care including lack of access to facilities, lack of time and money, and dependence on men for permission to leave the home, resulting in women either ignoring their problem, using self-care or self medication, using herbal or traditional medicine, or using services in secret [8-13]. Consultations with husbands, relatives and friends would also delay treatment-seeking for women, with prompt care triggered by symptoms that were perceived to be more severe or life threatening [14].”  I found this paragraph really interesting, as it contrasted with my assumptions and experiences regarding engagement with healthcare. The referenced articles support reduced levels of care-seeking in women in the region, however there is also a significant evidence base that supports that male engagement with healthcare is particularly problematic (this is often in the context of the HIV care cascade, e.g. described in the UNAIDS report “Male engagement in HIV testing, treatment and prevention in Eastern and Southern Africa” and other literature)  This is not essential, but the authors could consider briefly mentioning that, at least in other contexts, poor male engagement with healthcare is often more prominent. Could this perhaps have contributed to the lack of a gender difference in treatment-seeking? | Thank you for this suggestion and reference. We agree that male engagement in HIV care has been shown to be problematic and the factors that affect HIV and STI treatment-seeking are likely similar. We have revised the paragraph and added a sentence to illustrate factors that may affect treatment-seeking among men. | Lines 98-112  **Previous studies in the region have also observed lower levels of STI treatment seeking in women, with** barriers to care including lack of access to facilities, lack of time and money, and dependence on men for permission to leave the home, which resulted in women either ignoring their problem, using self-care or self-medication, using herbal or traditional medicine, or using services in secret [8-13]. Consultations with husbands, relatives and friends also delayed treatment seeking for women, with prompt care triggered by symptoms that were perceived to be more severe or life threatening [14]. **Notably, gender disparities in STI care stand in contrast to what is typically observed for HIV care and treatment where men are less likely to engage in HIV services than women [15].** |
| 2. Lines 121-122: “The RCCS includes the administration of a demographic and health questionnaire, as well as HIV testing for all consenting participants.”  A very minor point, but consider including how often each follow-up round is in the RCCS. | Thank you for this suggestion. We have added these details in the text. | Lines 132-133  The RCCS includes the administration of a demographic and health questionnaire, as well as HIV testing for all consenting participants, **every 18 to 24 months**. Details of the RCCS study design can be found elsewhere [17]. |
| 3. Lines 175-176: “For the remaining analyses, we restricted our sample to only those who reported any STI-related symptoms in the past six months (n=962).”  For clarity, please consider stating whether this includes or excludes participants who only reported “recent symptoms” in the past 7 days, as either option is arguably valid. | Thank you for the suggestion. We have added further clarification on this point in the text. | Lines 192-193  For the remaining analyses, we restricted our sample to only those who reported any STI-related symptoms in the past six months, **including those reporting symptoms in the past 7 days**. |
| 4. Lines 204-208: “Based on a conceptual framework of treatment-seeking behavior [1], we included in our model the number of STI-related symptoms experienced in the past six months, as well as select sociodemographic characteristics (age, community type, marital status, HIV status) that could theoretically affect treatment-seeking behavior.”  The reference for the conceptual framework is from a 1997 paper by Ward et al. The main model described in the paper by Ward et al. is the “determinants model”, itself described by Kroeger in 1983. Given how old these references are, I am wondering if any more up to date references could be provided that guided your choice of model variables. Additionally, a visual depiction of your framework/pathway would be really helpful, perhaps as a supplementary appendix? | Thank you for the suggestion. We have added our conceptual framework as a supplementary file (S1 Fig1). | Lines 222-233  **Informed by Kroeger’s determinants model [1, 20] and McLeroy et al.’s socioecological framework [21], we created a conceptual framework for treatment-seeking behavior (S1 Fig 1). Using this framework and prior literature, we selected variables for inclusion in the final multivariable models. Selected variables included** the number of STI-related symptoms experienced in the past six months, as well as select sociodemographic characteristics (age, community type, marital status, HIV status). All data analysis was carried out in STATA version 15 [22].  **S1 Fig 1, Conceptual framework. Informed by Kroeger’s determinants model and McLeroy et al.’s socioecological framework. This conceptual framework was developed to guide statistical analyses. Variables selected for inclusion in the multivariate model are underlined.** |
| 5. Lines 217-219: “Most men were aged 30-39 years, in a monogamous marriage, from the fishing community, Christian, educated at some level and working in the fishing industry.” & Table 1  A definition of “educated at some level” would be helpful. For example, does one year of schooling count as “educated at some level” or completion primary school or another cut-off? | Thank you for this suggestion. In response to Reviewer 1’s overarching comment to make the results more concise, we have edited the two sentences that referred to education in this way to remove mention of education and other variables that were not associated with treatment seeking (specifically, religion and occupation). In all tables, including Table 1, we changed the language to clarify that education was categorized as “some primary education or above” versus “no education”. | Lines 243-249  Table 1 summarizes the demographic characteristics of STIPS participants reporting STI symptoms. Most males were aged 30-39 years, in a monogamous marriage **and** from the fishing community**~~, Christian, educated at some level and working in the fishing industry~~**. Most females were aged 20-29 years **and also** in a monogamous marriage **~~and from the fishing community, Christian, educated at some level and working in agricultural or housework~~**.  We also have updated the text describing the education variable in all Tables. |
| 6. Table 1: Condom use  Was condom use asked of married people, or is it assumed that condoms were not used in marriage? If the former, I suggest providing this information. If the latter, please state this in the methods. | Thank you for this comment. Consistent with other research from the Rakai Community Cohort Study, we did not evaluate condom use in in marital relationships (it is rare) and focused on consistency of condom use in non-marital relationships. We have included a statement on this in the methods. | Lines 214-216  Complete treatment seeking information (i.e. any treatment seeking [yes or no] and specific treatment location) was collected for 99.4% of the sample, with only 6 participants dropped from the analysis because of missing treatment data. Age was analyzed in five-year age groupings. **Consistent condom use was assessed within non-marital relationships only; marital relationships were excluded because condom use is rare within these partnerships.** |
| 7. Lines 265-268: “Comparing private and government clinics, women were less likely to seek treatment at private clinics (more likely to seek government clinics) than men (PRR: 0.76, 95% CI: 0.68-0.85). This trend was seen in both the fishing (PRR: 0.86, 95% CI: 0.76-0.96) and inland (PRR: 0.65, 95% CI: 0.51-0.83) communities. When comparing men across communities, men in fishing communities were significantly more likely to seek treatment at a private clinic than men in inland communities (PRR: 1.25, 95% CI: 1.04-1.51). Similarly, women in the fishing community were significantly more likely to seek treatment at private clinics than women in the inland community (PRR: 1.65, 95% CI: 1.36-2.01).”  There is a huge amount of information in this paragraph, and one has to really concentrate to process it. I think the two main points are:  1. Women are more likely to seek treatment at government clinics than men (both communities) 2. Individuals in fishing communities are more likely to seek treatment at private clinics than in inland communities (both men and women)  Please consider condensing or re-wording this paragraph to be more concise. | Thank you for this suggestion. We have reworded this paragraph so that it more clearly addresses the main points, as well as added a supplementary table (S2 Table) to describe the results. | Lines 297-310  **S2 Table describes treatment seeking at private or government clinics by gender and community type. Females were more likely to seek treatment at government clinics) than males in both communities (S2 Table). Individuals in the fishing community were significantly more likely to seek treatment at a private clinic than those in the inland community (S2 Table). ~~Comparing private and government clinics, women were less likely to seek treatment at private clinics (more likely to seek government clinics) than men (PRR: 0.76, 95% CI: 0.68-0.85). This trend was seen in both the fishing (PRR: 0.86, 95% CI: 0.76-0.96) and inland (PRR: 0.65, 95% CI: 0.51-0.83) communities. When comparing men across communities, men in fishing communities were significantly more likely to seek treatment at a private clinic than men in inland communities (PRR: 1.25, 95% CI: 1.04-1.51). Similarly, women in the fishing community were significantly more likely to seek treatment at private clinics than women in the inland community (PRR: 1.65, 95% CI: 1.36-2.01).~~** |
| 8. Lines 302-306: “Just over half of men who reported seeking care at a clinic for their symptoms reported no symptoms in the past week (73/136 [54%]). In contrast, the majority of women who reported seeking care at a clinic for their symptoms reported having at least one STI symptom in the past week (66%), with 65/321 [20%] reporting one symptom 97/321, [30%] reporting 2-4 symptoms and 49/321 [15%] reporting five or more symptoms.”  Minor point: Consider re-phrasing to present the proportion of men who reported seeking care who reported at least one symptom in the past week, as this is how the equivalent statistic is presented for women. i.e. “Just under half of men who reported seeking care at a clinic for their symptoms reported at least one symptom in the past week…” This should make comparability between men and women slightly easier, without having to consider the inverse for men. | Thank you for this suggestion, we agree and believe this the manuscript is stronger because of it. We have rephrased the sentence so the comparison is clearer. | Lines 365-377  **Among those who sought clinical care for their symptoms in the last six months, 46% of males (63/136) and 66% of females (211/321) reported having at least one STI symptom in the past week. Furthermore, among ~~Just over half of men who reported seeking care at a clinic for their symptoms reported no symptoms in the past week (73/136 [54%]). In contrast, the majority of women who reported seeking care at a clinic for their symptoms reported having at least one STI symptom in the past week (66%), with 65/321 [20%] reporting one symptom 97/321, [30%] reporting 2-4 symptoms and 49/321 [15%] reporting five or more symptoms. When considering only those~~** males and females who reported at least one symptom in the past week, **~~we saw that, for both men and women,~~** just over half reported that they did not seek treatment at a clinic in the past six months for their symptoms (85/148 [57%] and 244/455 [54%], respectively). **~~We found no significant differences in any of these associations in stratifications by both gender and community type.~~** |
| 9. Table 2  This table provides lots of information. However, as the “clinic” and “no clinic” columns are the inverse of each other, you could consider removing one of these columns so that the table is more concise, and a bit less overwhelming. | Thank you for this suggestion. We have removed the ‘No Clinic’ column from Table 2. | Lines 359-364 (Table 2) |
| 10. Table 2  Minor point – For “Number of STI symptoms in past 6 months” for males, the odds ratio for 2-4 is 1.72 (1.34 – 2.21), but there is no associated ** or *** | Thank you for this note, we have reviewed Table 2 and adjusted the text appropriately. | Lines 359-364 (Table 2) |
| 11. Lines 340 – 342: “In multivariable analyses, significant factors associated with seeking treatment at a clinic for men included being from the inland community and having multiple STI-related symptoms in the past six months.” & Table 3  Please see previous comment regarding the conceptual framework. More information needs to be provided in the methods on the conceptual framework and how that led you to decide to include or exclude variables. | Thank you for this comment. We have added our conceptual framework as a supplementary file (S1 Fig1). | See S1_Fig1.tif |
| Table 4  Similar comment re: table 2 about redundancy of some data presented. As “yes” and “no” for “any curable STI” are the inverse of each other, I suggested removing the “no” column. | Thank you for the suggestion. We have removed the ‘No’ column from the table. | Lines 432-437 (Table 3) |
| ***Reviewer #2*** |  |  |
| The study by Ogale et al. reports on treatment seeking behaviours for sexually transmitted infections in rural communities from Uganda. The authors report that while STI symptoms are common, a considerable proportion of people do not seek treatment from formal healthcare (or at all). In addition, among those with STI symptoms the prevalence of STIs was very high. This study is very relevant to understanding the context for STIs in this setting (and likely more generally in communities across sub-Saharan Africa) and can contribute to developing strategies for improving SRH services. | Thank you for the review! |  |
| Methods: study setting: - please add the catchment population size to provide more context to the data | Thank you for the suggestion. We have added more details on the underlying census eligible population in the methods. | Lines 147-149  STIPS was a population-based survey that recruited all individuals aged 18–49 years from two RCCS communities—one inland and one fishing—from May to October 2019 [16]. These communities were selected because of their representativeness, geographical diversity, population size, and enrollment timelines within the RCCS survey. Three adjacent villages consisting of semi-urban and rural agrarian populations comprised the inland community and one fish landing site along Lake Victoria comprised the fishing community. **There were 2583 census-eligible participants, of whom 1884 were present at the time of RCCS survey, 1877 consented to participate in RCCS survey and 1825 consented to participate in STIPS.** |
| - describe how the 2 communities included in this study were selected from the entire population. | Thank you for this suggestion. We have added a sentence describing how the communities were selected. | Lines 143-145  STIPS was a population-based survey that recruited 1825 individuals aged 18-49 years from two RCCS communities – one inland and one fishing – from May to October 2019 [16]. **These communities were selected because of their representativeness, geographical diversity, population size, and enrollment timelines within the RCCS survey.** Three adjacent villages consisting of semi-urban and rural agrarian populations comprised the inland community and one fish landing site along Lake Victoria comprised the fishing community. |
| Results: - a flow diagram reporting on inclusion would be helpful in providing an overview of people eligible to participate, those included, those interviewed, and those tested for STIs. This can be added in the supplement | Thank you for this suggestion. Because a detailed flow diagram reporting inclusion in the STIPS was provided in the main STIPS paper (reference number 16), we have opted not to include the same figure in this manuscript. However, we have added additional text to describe the census eligible population. | Lines 147-149  STIPS was a population-based survey that recruited all individuals aged 18–49 years from two RCCS communities—one inland and one fishing—from May to October 2019 [16]. These communities were selected because of their representativeness, geographical diversity, population size, and enrollment timelines within the RCCS survey. Three adjacent villages consisting of semi-urban and rural agrarian populations comprised the inland community and one fish landing site along Lake Victoria comprised the fishing community. **There were 2583 census-eligible participants, of whom 1884 were present at the time of RCCS survey, 1877 consented to participate in RCCS survey and 1825 consented to participate in STIPS.** |
| Discussion - The discussion could be improved by commenting on people with STIs who are asymptomatic and who contribute to transmission (given that a considerable proportion of people who have positive tests for STIs are in fact asymptomatic) and on the importance of the availability of diagnostics for STIs given the high prevalence in these communities and the limitations of syndromic management. The latter is only very briefly mentioned in the conclusion and would deserve a somewhat more extensive comment. If possible it would be interesting to also comment on the prevalence of STIs (positive tests) in Uganda or in other countries from sub-Saharan Africa to provide more context to the high prevalence reported here. | Thank you for this comment. We very much appreciate these points, which are discussed in further detail in the main manuscript from the STIPS trial which talks about asymptomatic STIs in depth. We now cite this paper (reference 16) and have added a few details to the discussion and conclusion to highlight asymptomatic individuals and that more can be done to address STIs in the community. As our study focused on treatment-seeking, which is less relevant for asymptomatic STIs, we feel a reference to the main study manuscript here is most appropriate for individuals interested in further exploration of these issues. | Lines 543-544  We also found that approximately one-third of males and females who previously sought clinic treatment for their symptoms were diagnosed with at least one curable STI (CT, NG, TV or active syphilis) at the time of the survey. Furthermore, our analysis showed no difference in the current prevalence of curable STIs comparing those who previously sought clinic treatment versus those who did not, for either sex. Assuming that seeking clinic treatment meant receiving treatment, these data could indicate that reinfection rates were high, treatment was inadequate, or both. **A discussion on STI prevalence and asymptomatic infection in STIPS communities regardless of treatment seeking is explored elsewhere [16];** further studies exploring the temporal association between past treatment seeking, including receiving and adhering to treatment, and current STI prevalence are recommended to assess treatment effectiveness.  Lines 604-608  Timely and appropriate diagnosis is critical to STI treatment and control. We found that half of adults with STI symptoms in two rural Ugandan communities are not seeking appropriate clinical care under the syndromic management strategy. We recommend researchers continue to focus on treatment-seeking behavior in low-resource settings and explore barriers to seeking care, including health system barriers such as low quality of care. **However, as many people with STIs are asymptomatic in this and other settings, we also urge decision-makers to increase support for STI services, as well as increased availability of diagnostics, in this and similar contexts.** |
| - Another point that can be discussed is that for women (who reported symptoms more frequently than men) symptoms considered as suggestive of STIs included lower abdominal pain, dysuria, and frequency. These symptoms can also be due to urinary tract infections which are common in women. | Thank you for the comment. We have added a sentence in the discussion that considers the overlap between UTI and STI symptoms. | Lines 495-498  A similar phenomenon may have occurred in our sample: participants may not have attributed lower abdominal pain, warts or discharge to an STI, thereby explaining why the presence of some symptoms were associated with clinic treatment over others. **For females, symptoms such as lower abdominal pain, dysuria, and frequent urination can also be due to urinary tract infections, which are common among females, and which may help explain why females reported symptoms more often than males in our study.** |
| - It would also be interesting to comment on gender differences in terms of number of sexual partners (women having fewer) and types of sexual partners (more likely to be from inside the community). | Thank you for the comment. We have added a sentence in the discussion that addresses this point and cites several other studies from the Rakai Community Cohort Study, which have delved into this issue in more depth (references 35 and 36). | Lines 459-462  While the 2016 Uganda DHS estimates show a higher prevalence of clinic treatment-seeking among females than males [5], as aforementioned, we are hesitant to compare our findings to DHS estimates because of urban/rural differences. **We also found that sexual behaviors which may influence STI risk, such as having multiple partners and types/locations of sexual partners (i.e. household, community, extra-community partners), differed by sex, as has been explored further in other publications from the RCCS [35, 36].** |
| Tables and figures: Figure 2: consider writing “private” in full rather than abbreviated. | Thank you for this suggestion, we have updated the figure so “government clinic” and “private clinic” are written in full. | See S1_Fig1.tif |
| Abstract line 61 – missing „for“ | Thank you for catching this. We have added the missing word. | Line 63  In this setting, nearly half of adults with STI-related symptoms are not seeking clinical care and many who report having sought treatment **for** recent STI symptoms have curable STIs. |
